# Host restriction factor SAMHD1 limits human T-cell leukemia virus (HTLV-1) infection of primary monocytes via the innate immune sensor STING

**DOI:** 10.1186/1742-4690-11-S1-O19

**Published:** 2014-01-07

**Authors:** Alexandre Sze, S Mehdi Belgnaoui, Rongtuan Lin, Julien van Grevenynghe, John Hiscott

**Affiliations:** 1 Lady Davis Institute-Jewish General Hospital, McGill University, Montreal, Quebec, Canada; 2 Vaccine and Gene Therapy Institute of Florida, Port St. Lucie, FL, USA

## 

Human T-lymphotropic virus type 1 (HTLV-1) is the causative agent of adult T cell leukemia (ATL) and other HTLV-1 associated neurological disorders. Unlike most retroviruses, cell-free HTLV-1 virions are poorly infectious and do not stably infect its primary CD4+ T lymphocyte target. However, HTLV-1 efficiently infects cells of the myeloid lineage, leading to productive infection of myeloid cells. Here, we investigate the mechanisms underlying monocyte infection by HTLV-1 and demonstrate that HTLV-1 infection induced apoptosis of monocytes in a SAMHD1-dependent manner. SAMHD1, a deoxynucleoside triphosphate triphosphohydrolase, functions as a restriction factor that limits HIV-1 replication by reducing the availability of deoxynucleosidetriphosphates (dNTPs) required for reverse transcription. RNAi-mediated silencing of SAMHD1 inhibited monocyte apoptosis, while addition of exogenous dNTPs, or pre-treatment with azidothymidine (AZT) to block reverse transcription also inhibited apoptosis. To investigate a role for reverse transcription intermediates (RTI) in triggering apoptosis, a biotinylated 90 nucleotide RTI from the U5 region of HTLV-1 was introduced into monocytes; strikingly, the biotinylated RTI induced apoptosis and bound to the DNA sensor STING - which mediates the antiviral response via IRF3 activation. We further demonstrated that STING-mediated apoptosis in infected monocytes required the generation of a pro-apoptotic complex between IRF3 and the Bcl-2 protein Bax. These studies provide a mechanistic explanation for HTLV-1 abortive infection of monocytes and report a link between SAMHD1 restriction of reverse transcription, sensing of retroviral reverse transcription intermediates by STING, and the initiation of IRF3-Bax driven apoptosis.

